# 3D convolutional neural network for machining feature recognition with gradient-based visual explanations from 3D CAD models

**DOI:** 10.1038/s41598-022-19212-6

**Published:** 2022-09-01

**Authors:** Jinwon Lee, Hyunoh Lee, Duhwan Mun

**Affiliations:** School of Mechanical Engineering, Korea University, 145, Anam-ro, Seongbuk-gu, Seoul, 02841 Republic of Korea

**Keywords:** Aerospace engineering, Mechanical engineering

## Abstract

In the manufacturing industry, all things related to a product manufactured are generated and managed with a three-dimensional (3D) computer-aided design (CAD) system. CAD models created in a 3D CAD system are represented as geometric and topological information for exchange between different CAD systems. Although 3D CAD models are easy to use for product design, it is not suitable for direct use in manufacturing since information on machining features is absent. This study proposes a novel deep learning model to recognize machining features from a 3D CAD model and detect feature areas using gradient-weighted class activation mapping (Grad-CAM). To train the deep learning networks, we construct a dataset consisting of single and multi-feature. Our networks comprised of 12 layers classified the machining features with high accuracy of 98.81% on generated datasets. In addition, we estimated the area of the machining feature by applying Grad-CAM to the trained model. The deep learning model for machining feature recognition can be utilized in various fields such as 3D model simplification, computer-aided engineering, mechanical part retrieval, and assembly component identification.

## Introduction

The smart factories control the production automation, process plan, physical distribution, and services in an integrated way by fusing IT systems with traditional manufacturing industries^1^. For factories of manufacturing industries to be converted into smart factories, all the things that are manufactured must be managed using computer-aided design (CAD). The three-dimensional (3D) CAD model of the boundary representation (B-rep) form widely used in the field includes geometric information (for example, NURBS, cylinder, and circle) that expresses shapes and topology information (for example, faces, edges, and vertices) that expresses the neighborhood relationship between geometric elements^2^. Although it is easy to express the design result of a product using such a 3D CAD model, it is not suitable as a computer-aided manufacturing model for physical products. For a cutting work that processes a part by cutting material, a set of high-level machining features such as slots, holes, and chamfers, not a B-rep model comprising geometric or topology information, are required as input data. In this study, the focus was on the recognition of different machining features in a 3D CAD model.

Studies on feature recognition methods have been actively conducted for 40 years^3^ in both the industrial world and academic^4,5^. Although various conventional methods are introduced for machining feature recognition, there was a limitation that recognition was only possible under certain conditions. Recently, there have been various attempts to apply deep learning technology to the 3D CAD field^6–8^. FeatureNet utilized the voxel dataset and 3D convolutional neural networks to recognize machining features^9^. They recognized single machining features with 96.7% accuracy, but multi-features, where more than one feature exists in a single model, could not be distinguished. In addition, they only could classify whether the machining features were included and could not estimate area of the feature.

In this paper, we propose a novel 3D convolutional neural network (3D-CNN) to recognize machining features and to detect feature areas from a 3D CAD model. A CAD model that includes single- and multi-features is converted into a voxel, and then the machining features are classified using a 3D-CNN. To improve the accuracy of classification, we utilize various network methods that have not been applied in other studies; (1) applying general conv layer- pooling layer in the voxel network; (2) adjusting network hyperparameter such as 1 stride set to the first conv layer; (3) using 1 × 1 conv filter as the last layer; (4) using the global average pooling layer instead of fully connected layer. In addition, the feature area of each model is estimated by applying class activation mapping without an additional network model that utilizes a bounding box annotation.

The proposed network is well designed using several deep learning techniques to achieve the goal of recognizing single- and multi-machining features in 3D voxels, which clearly shows the combinational novelty of this study. The specific contributions of this study are as follows: (1) a novel network to classify the machining features with high accuracy; (2) feature area detection by analyzing the gradient of the network. In particular, gradient-weighted class activation mapping (Grad-CAM) is applied to 3D voxels to recognize machined features area detection; (3) constructing 3D CAD datasets with machining features for deep learning; (4) simultaneous training and classification of single- and multi-features, in contrast to the existing studies that have trained only single features; and (5) a deep learning model capable of being expanded to various fields such as 3D model simplification, computer-aided engineering, mechanical part retrieval, and assembly component identification.

This paper is organized as follows: “Related works” section introduces the studies related to feature recognition and deep learning-based area detection. “Data generation and preprocessing” section describes the data generation and conversion processes used for network learning. “3D-CNN for machining feature classification” section proposes a new form of 3D-CNN, and “Class activation mapping for feature area detection” section describes the method for estimating feature areas using class activation mapping. “Experiments and discussion” section analyzes the results of the feature recognition classification and feature area detection performed in the experiment. In Sect. 7, we conclude with a summary of this study and provide the potential for future work.

## Related works

### Conventional 3D feature recognition

Conventional methods for recognizing the features of 3D CAD models can be divided into graph-based, volume decomposition, hint-based, and similarity-based methods^10^. The graph-based method expresses the relationship between a face and an edge in a graphic structure and performs recognition by analyzing whether the graph of the model corresponds to the specific pattern of a feature. Elinson et al. evaluated the similarity by expressing the relationship of machining features in a graph^11^. However, as certain simple forms of machining features for milling and drilling were considered as the targets of similarity comparison, it was difficult to apply them to complicated shapes. In addition, Kim et al. proposed a wrap-around algorithm that removes a feature by expanding the faces adjacent to the feature or generating a new face and a smooth-out algorithm that simplifies the model by removing the features that have relatively small volumes among the features that comprise the model^12^. The similarity was compared after changing the complicated shape model that was input to a base feature using this algorithm. Although computation may be reduced if the similarity is compared after changing the features to simple base features, the accuracy deteriorates because the major features of the shape model that have been entered are removed.

The volume decomposition method decomposes a complicated model into different types of simple volumes and feature-recognizes simple shapes. Woo proposed fast volume decomposition (FVD) to improve the existing cell-based decomposition method, which requires significant time for calculations^13^. They generated volumes after conducting cellular decomposition by localized face extension and cell collection using seed cells. Kim and Mun proposed a non-overlapping volume decomposition method that minimizes the overlap of the volumes decomposed from a solid model^14^.

The hint-based method defines the hint of a feature in the object to be recognized and determines the feature using the geometric inference of the shape that corresponds to the hint. It was proposed by Vandenbrande and Requicha for the first time, and an object-oriented feature finder (OOFF) was introduced to determine the hint from the faces of slots, holes, and pockets^15^. Han and Requicha proposed an incremental feature finder (IF2) developed by improving the function of the OOFF^16^.

The similarity-based method determines features by evaluating the similarities between the two models. Hong, Lee, and Kim generated a high-resolution model and a low-resolution model from a 3D CAD model comprising B-rep using multi-resolution modeling and then used the low-resolution and high-resolution models to compare the overall shape and detailed shape of the model, respectively^17^. Ohbuchi et al. evaluated the similarities by generating the distances of the model areas distributed in reference to the axis of rotation as a histogram using the moment of inertia^18^. However, this method exhibited a good similarity recognition rate only when the shape was symmetrical. Jeon et al. extracted the features of a shape model that exhibited a geometric nature and evaluated the similarity between an existing shape model and a new model using the probability distribution histogram generated using this feature^19^.

### Deep learning-based 3D feature recognition

As CNNs have exhibited excellent performance in 2D image classifications, researchers have begun studies on using deep learning for 3D feature classifications. A 3D CAD model comprising irregular and unordered point clouds was converted into a standardized voxel grid, which was then classified using a 3D convolution filter^6,7^. Maturana and Scherer designed a network in such a way that it is rotationally invariant to allow the voxel data to maintain a consistent orientation^20^. For this, they augmented the training dataset by performing the rotation of the model. Qi et al. classified a model by combining two types of networks^21^. The first network prevented overfitting by employing the structure of simultaneously learning the whole and a part of a 3D model using the multilayer perceptron convolution (MLPConv) layer, and the second network exhibited an effect similar to that of the multi-view method by projecting 3D voxels onto a 2D image using the end-to-end method.

Recently, researchers have conducted studies on recognizing the machining features in a stock model. Zhang et al. proposed FeatureNet, which utilizes a 3D-CNN to determine the machining features of a machine part^9^. They generated a dataset comprising 24 machining features, such as holes, slots, steps, chamfers, and rounds, which were converted into voxels of a fixed size to train a network. They achieved a classification accuracy of 97.4% when the voxel resolution was 64. Peddireddy et al. learned feature recognition with a 3D-CNN to identify the machining process in a CAD model and expanded the trained network using deep transfer learning^22^. Nine milling features and seven drilling features were used for feature recognition, and it exhibited a classification accuracy of 93.47% after learning 20,000 epochs. However, the classification accuracy of the validation dataset deteriorated owing to excessively trained overfitting in the training dataset. Shi et al. generated a feature based on a heat kernel signature and expressed the feature as a graph using a percentage similarity clustering technique and a node embedding technique^23^. Subsequently, they recognized the feature by applying a 2D-CNN based on the graph. Ning et al. proposed a method of recognizing 14 features using a 3D-CNN and a method of establishing the relationship between quantity and cost by expressing the identified feature in quantity^24^. They applied a support vector machine (SVM) and back-propagation (BP) networks to establish the relationship between quantity and cost. Nie et al. proposed a new network that employs a deep multi-attention network for multi-view images^25^. They were able to efficiently extract information by considering the correlations among multi-views in an attention network. Certain studies captured images while rotating a 3D model at different angles as input data for feature recognition^26,27^. After learning the 2D image neural network based on the captured images, the information obtained at different angles was combined. Ghadai et al. applied a 3D-CNN to classify manufacturability in a model comprised of drilled holes^28^. They augmented the voxel data in the normal vector direction of the object boundary to augment insufficient data. A comparison between our method and previous works on deep learning-based feature recognition is shown in Table 1.

**Table 1:** Comparison between our method and related works.

Research category	Related works		Our method
Deep learning- based feature recognition	^9^	Machining feature classification with 3D-CNN for single feature Networks consisting of 7 layers (4 conv, 1 pooling, and 2 fully connected layers) Releasing open dataset for machining feature consist of 24 categories	Machining feature classification for single- and multi- features Networks consisting of 11 layers (5 conv, 3 pooling, 1 dropout, 1 GA, and 1 fully connected layers) Applying general conv-pooling block in the voxel network To minimize the loss of information, 1 stride set to the first conv layer Using the 1 × 1 conv filter as the last conv layer Using the global average pooling layer to prevent overfitting
	^28^	Manufacturability analysis of drilled holes with deep learning Networks consisting of 4 layers (2 conv, 1 pooling, and 1 fully connected layers)	
	^22^	Classification of nine milling features and seven drilling features Networks consisting of 6 layers (3 conv, 1 pooling, and 2 fully connected layers) Transfer learning applied to use the layer trained for feature classification as the machining process recognition layer	
	^23^	Manufacturing feature recognition with 2D-CNN Networks consisting of 4 parallel conv-polling blocks (for each, 2 conv, 2 pooling, and 2 dropout layers) Mesh converted into graph data using heat kernel for use as input to 2D-CNN	
Explainable deep learning	^31^	Extracting the area of the object in the feature map with class activation mapping Obtaining the localization feature by using global average pooling (GAP)	Applying the Grad-CAM for 3D voxels Estimating the area of single- and multi-feature Extracting the visual explanation for multi conv layers
	^32^	Applying gradient-weighted class activation mapping (Grad-CAM) for networks that don’t have GAP Extracting the visual explanation for conv layer regardless of GAP	
	^34^	Improving the network performance with visual explanation Introducing attention mechanism with the result of class activation mapping as input	

### Class activation mapping

As the studies that used deep learning exhibited good performance in the image field, researchers attempted diverse studies to interpret the basis on which networks are trained. However, most studies have been conducted only at the level of the filter, such as visualizing each filter or determining the input that has the maximum activation in a specific filter^29,30^. Zhou et al. proposed class activation mapping that obtains localization by using global average pooling (GAP) even when there is only a label for classification, with no label information for each pixel^31^. They identified the rationale for explaining the selection of the area on which the network performed classification by conducting the matrix multiplication of the feature maps before performing each weight and GAP in softmax, the last layer, and then combining them. They accomplished 37.1% (based on top-5) for the object location detection of the ImageNet Large Scale Visual Recognition Competition (ILSVRC) 2014 with no pixel information. Models did not have GAP cannot be utilized in the existing class activation mapping. To resolve this problem, Selvaraju et al. proposed Grad-CAM that uses the gradient of back-propagation^32^. Chattopadhay et al. changed the gradient generation process in such a way that areas were estimated irrespective of the estimated object size to improve the situation where Grad-CAM identified only certain areas without covering the entire area of the object^33^. Fukui et al. applied visual explanation obtained using class activation mapping to improve network performance^34^. They created an attention map with a feature map at the attention branch using a method similar to that of class activation mapping and predicted class probability with the feature map and the attention map at the perception branch using an attention mechanism. Omeiza et al. tried to alleviate saliency map noise and visual diffusion by adding noise to the inputs^35^. However, such maps are low in quality and typically have considerable noise. A comparison between our method and previous works on explainable deep learning is shown in Table 1.

### Data generation and preprocessing

To train models using deep learning, datasets that include machining features are required. In some studies using 3D-CNN to classify machining features, datasets with only single features were considered^9,22^. However, datasets with only single machining features are difficult to learn information for multi-features and, thus, the accuracy for multiple features was low.

**Figure 1:**
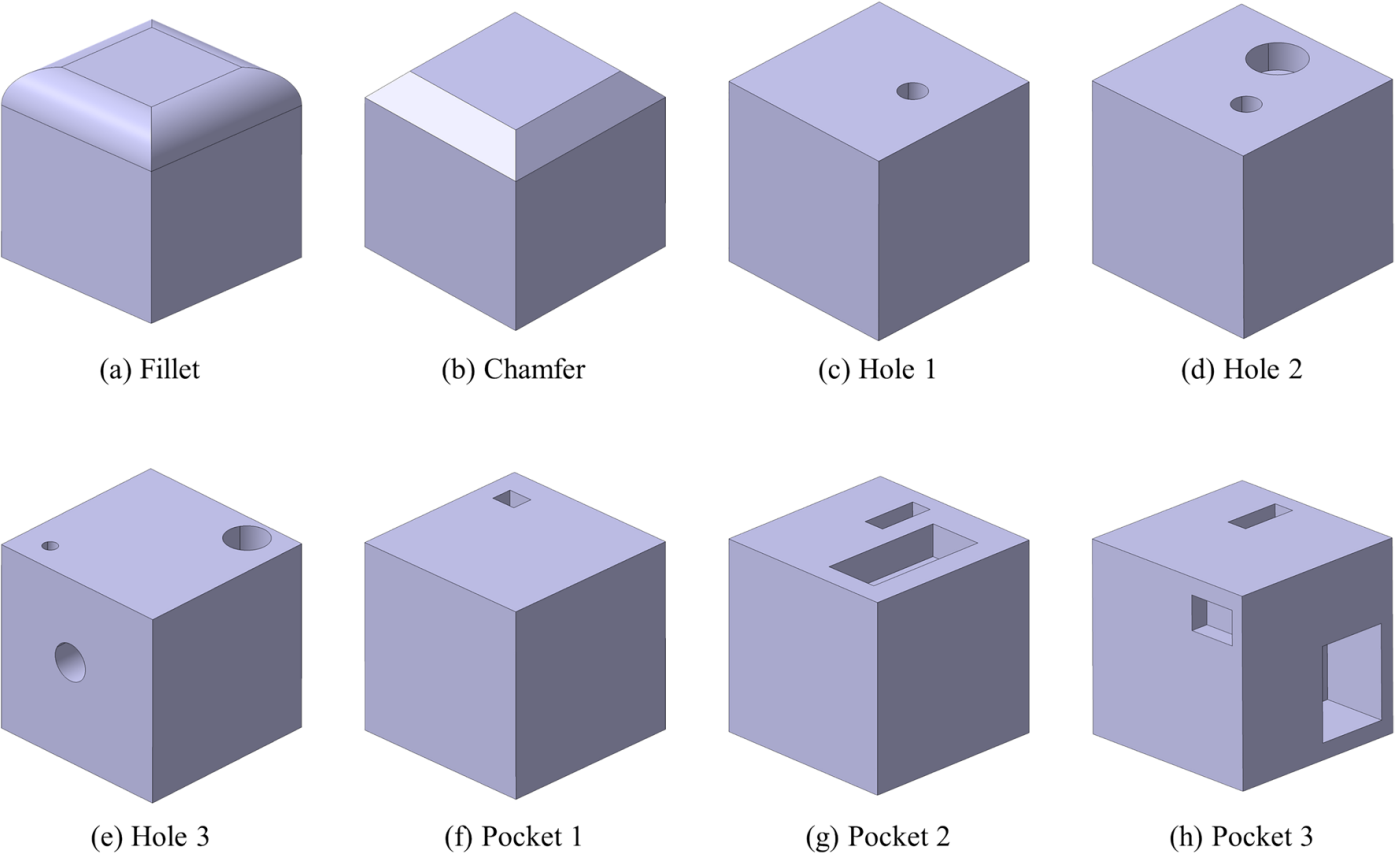
Types of machining features used in our study.

In this study, we construct a dataset consisting of single feature and multi-feature (multi-instance of a single feature, multi-instances of multi-feature). Figure 1 shows the four single features and four multi-instances of single features defined in this study. The single features selected were the basic and widely used hole, pocket, fillet, and chamfer. Multi-instance of a single feature means that multiple holes and pockets are duplicated in one model. Multi-instance of multi-feature represents a combination of single feature and multi-instance of a single feature. Whereas the fillet and chamfer cannot be included simultaneously in a model, the hole and pocket are arranged to be included simultaneously in a model.

**Figure 2:**
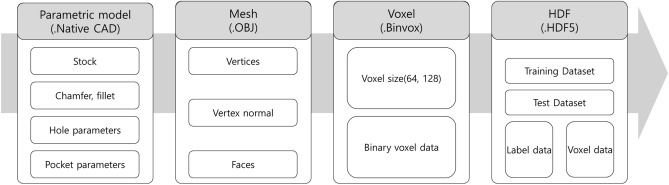
3D CAD model generation and conversion processes.

Figure 2 presents the data generation and conversion processes from a commercial 3D CAD system (Dassault Systèmes’ CATIA) to HDF5^36^, a data format for machine learning. Parametric modeling is used to create many 3D CAD models with machining features. Table 2 presents the parameters used for model generation. Values are randomly selected as the parameter values within the predefined range. For example, to generate a model for which a hole and fillet are used, two Booleans (fillet and stock round) and eight parameters are sampled: ${S}_{w}$, ${S}_{h}$, ${S}_{d}$, ${S}_{r}$, $F$, ${X}_{h}$, ${Y}_{h}$, ${R}_{h}$, ${D}_{h}$, and ${O}_{h}$.

**Table 2:** Parameters used to control the shape of a 3D CAD model.

Type	Parameters		
Stock	Stock width (${S}_{w}$)	Stock height (${S}_{h}$)	Stock depth (${S}_{d}$)
	Stock round (${S}_{r}$)		
Edge	Fillet (${F}^{*}$)	Chamfer (${C}^{*}$)	
Hole	Hole x-coordinate (${X}_{h}$)	Hole radius (${R}_{h}$)	Hole orientation (${O}_{h}$)
	Hole y-coordinate (${Y}_{h}$)	Hole depth (${D}_{h}$)	
Pocket	Pocket x-coordinate (${X}_{p}$)	Pocket width (${W}_{p}$)	Pocket depth (${D}_{p}$)
	Pocket y-coordinate (${Y}_{p}$)	Pocket height (${H}_{p}$)	Pocket orientation (${O}_{p}$)

*Means Boolean parameter.

A CNN learns the spatial information of the structured data expressed in the form identifiable by a computer. For 3D CAD models expressed in B-rep, unlike 2D images, CNNs learning is difficult because of their complexity in element composition and relation amongst them, as well as non-uniform data size. Accordingly, they have to be converted to voxels of a 3D occupancy grid that are easy to learn the network. Voxels are created by extending the dimension of 2D pixels to 3D volumes, and it represents element with the same cube volume within a fixed grid. Voxels can be easily applied to a CNN because it is expressed as one if a voxel has a shape within the grid and zero if it has no shape.

The constructed dataset consists of 119,320 features; the number of single features is 56,100, and the number of multi-features consisting of two or more features is 63,220. Table 3 summarizes the amount of data in each dataset category. The table parentheses indicate the amount of data with fillets or chamfers applied. For example, there are 7020 multi-feature data with one hole and three pockets simultaneously, and 1020 of those include fillets or chamfers.

**Table 3:** The amount of data by category for the dataset.

	None	Hole 1	Hole 2	Hole 3
None	0	14,000 (2000)	14,100 (2100)	0
Pocket 1	14,000 (2000)	12,600 (2100)	12,000 (2000)	7000 (1000)
Pocket 2	14,000 (2000)	12,600 (2100)	12,000 (2000)	0
Pocket 3	0	7,020 (1020)	0	0

Data encoding methods include label encoding and one-hot encoding. Label encoding defines data sequentially by using integer-type values. This method deteriorates the prediction performance because the dependence between each class is ignored. One-hot encoding is a method that assigns a column corresponding to a unique value to one data type and marks the remaining columns with 0. This method is suitable for this study because the correlation between each class can be trained. We set multiple labels for the data. Table 4 presents the eight machining features which are expressed in one-hot encoding. Multi-features were generated by combining them through Boolean operations. For example, a 3D CAD model comprised of fillet, two holes, and two pockets is expressed as (1, 0, 0, 1, 0, 0, 1, 0).

**Table 4:** Definition of one-hot vector label for machining features.

Index	1	2	3	4	5	6	7	8	One-hot vector
Type									
Fillet	1	0	0	0	0	0	0	0	(1, 0, 0, 0, 0, 0, 0, 0)
Chamfer	0	1	0	0	0	0	0	0	(0, 1, 0, 0, 0, 0, 0, 0)
Hole 1	0	0	1	0	0	0	0	0	(0, 0, 1, 0, 0, 0, 0, 0)
Hole 2	0	0	0	1	0	0	0	0	(0, 0, 0, 1, 0, 0, 0, 0)
Hole 3	0	0	0	0	1	0	0	0	(0, 0, 0, 0, 1, 0, 0, 0)
Pocket 1	0	0	0	0	0	1	0	0	(0, 0, 0, 0, 0, 1, 0, 0)
Pocket 2	0	0	0	0	0	0	1	0	(0, 0, 0, 0, 0, 0, 1, 0)
Pocket 3	0	0	0	0	0	0	0	1	(0, 0, 0, 0, 0, 0, 0, 1)

### 3D-CNN for machining feature classification

**Figure 3:**
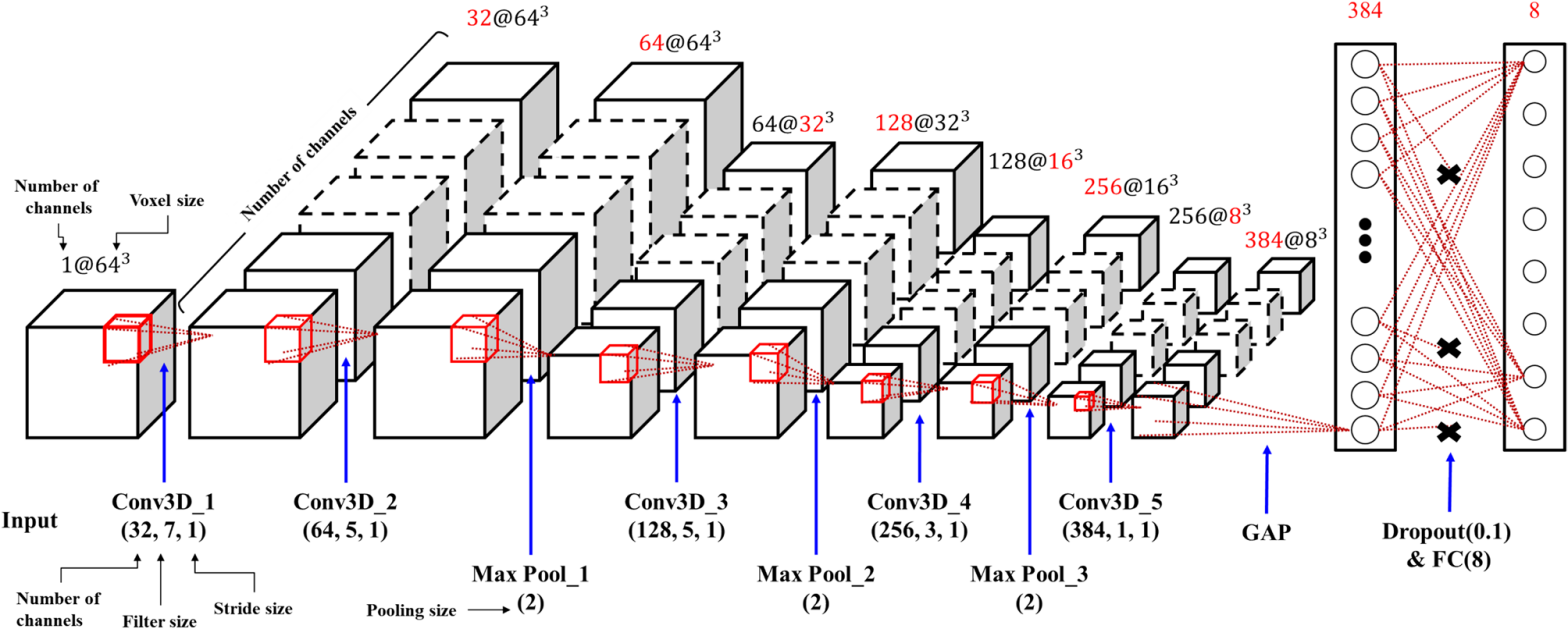
Network architecture for feature recognition.

For a long time, several researchers have been conducting studies to find machining features contained in CAD models. Conventional machining feature recognition has limitations in that it is difficult to generalize the method, and the recognition rate drops when the noise occurs with the data (interference between features). To resolve these issues, we propose a novel network for classifying the machining features in a CAD model using a 3D-CNN. Figure 3 depicts the proposed 3D-CNN architecture. The network includes 12 layers: one input layer, five 3D convolution layers, three max-pooling layers, one GAP layer, one dropout layer, and one fully connected (FC) layer. In each layer, the data size is indicated as (number of channels @ voxel size).

The input layer receives the data expressed in a voxel of a fixed size (I × J × K) as the input. The sizes of the input data used for the experiment were 64 × 64 × 64 (1@64^3^) and 128 × 128 × 128 (1@128^3^). The input layer is followed by convolution layers and max-pooling layers. The 3D convolution layers extract the features of the adjacent data by receiving 4D data (width, height, length, and channel) and multiplying them with a 3D convolution filter (width, height, and length). The parameters of the convolution layer are the number of channels, filter size, and spatial stride length. The product of the convolution filter passes through an activation function that changes it to a nonlinear value. A rectified linear unit (ReLU) that efficiently learns in a deep neural network is used as the activation function. The 3D max-pooling layer reduces the data size while maintaining the spatial features of the data and extracts only the largest value within the predefined filter size. The parameter of the max-pooling layer is a three-dimensional filter size (f).

In contrast to earlier studies^9,22^ that have used successive convolution layers and one max-pooling layer, we propose a network structure that increases the number of channels instead of gradually decreasing the voxel size. In addition, FeatureNet reduces the data size by using two as the stride in the first convolution layer^9^. If the size of the hole or pocket is small, the classification accuracy decreases as the voxel size decreases. In this study, we propose two methods to learn small-sized features. First, we placed max-pooling layers instead of using a stride of 2 in the first convolution layer. Second, Conv3D_5 used a 1 × 1 convolution filter to increase the nonlinearity of the deep learning model and allow spatial features to be well trained. Table 5 lists the parameter layers and input/output shapes we used after the input layer.

**Table 5:** Parameters and input/output shape of convolution layer max-pooling layer.

Layer name	Parameter	Input shape	Output shape
Conv3D_1	(32, 7, 1)	$1@{64}^{3}$ ($1@{128}^{3}$)	$32@{64}^{3}$ ($32@{128}^{3}$)
Conv3D_2	(64, 5, 1)	$32@{64}^{3}$ ($32@{128}^{3}$)	$64@{64}^{3}$ ($64@{128}^{3}$)
Max-pooling_1	(2)	$64@{64}^{3}$ ($64@{128}^{3}$)	$64@{32}^{3}$ ($64@{64}^{3}$)
Conv3D_3	(128, 5, 1)	$64@{32}^{3}$ ($64@{64}^{3}$)	$128@{32}^{3}$($128@{64}^{3}$)
Max-pooling_2	(2)	$128@{32}^{3}$ ($128@{64}^{3}$)	$128@{16}^{3}$($256@{32}^{3}$)
Conv3D_4	(256, 3, 1)	$128@{16}^{3}$ ($256@{32}^{3}$)	$256@{16}^{3}$($256@{32}^{3}$)
Max-pooling_3	(2)	$256@{16}^{3}$ ($256@{32}^{3}$)	$256@{8}^{3}$($256@{16}^{3}$)
Conv3D_5	(384, 1, 1)	$256@{8}^{3}$ ($256@{16}^{3}$)	$384@{8}^{3}$($384@{16}^{3}$)

The product of the Conv3D_5 layer sequentially passes through the GAP layer, dropout layer, and FC layer. The GAP layer transforms a feature into a 1D vector by extracting the average value of each feature map. As a smaller parameter is required when the GAP layer turns a feature into a 1D vector using the FC layer, it helps to prevent overfitting. The dropout layer prevents overfitting by blocking the signals from going to the next layer at a certain probability. The FC layer connects all input and output data. We used a sigmoid as the activation function of the FC layer to obtain the probability for each feature to exist. As the loss function was used to evaluate and minimize the current model, we used binary cross-entropy. Binary cross-entropy is used to classify two classes, True or False, and is expressed as in Eq. (1).

1 $$ L = - \frac{1}{N}\sum\nolimits_{i = 1}^{N} {t_{i} \log \left( {y_{i} } \right) + \left( {1 - t_{i} } \right)\log \left( {1 - y_{i} } \right)} $$

where ${t}_{i}$ denotes a one-hot encoded true label vector, and ${y}_{i}$ denotes the vector of the predicted probabilities from the FC layer.

### Class activation mapping for feature area detection

**Figure 4:**
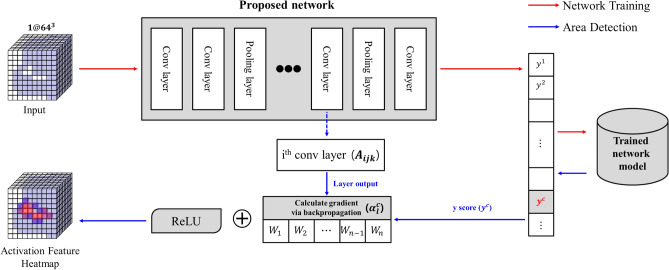
Feature area detection using Grad-CAM.

Deep learning networks exhibit superior performance in classifying the object types presented by input images, point clouds, and voxels. However, we do not know the computations used for classifications inside a deep learning network as it is similar to a black box. Therefore, studies on explainable AI (XAI) have been conducted to search for and interpret the basis of the prediction performed by deep learning models^31–33^. We intend to search for feature areas by interpreting the results predicted by networks using Grad-CAM. Grad-CAM interprets and visually explains deep learning models using a gradient, which is the weighted value of the filter in the back-propagation process of learning networks. Figure 4 demonstrates the process of tracing a feature area using Grad-CAM.

First, an intermediate output that has passed through the desired convolution layer is obtained. In addition, the y score (${y}^{c}$), which is the final output of a specific class ($c$) intended to be traced, is obtained. Equation (2) defines ${\alpha }_{l}^{c}$ Which is called a neuron importance weight, using these two types of information, where A denotes the convolution filter; i, j, and k denote the values in the filter corresponding to x, y, and z, respectively; and l denotes the number of filters. $\alpha$ is the weight that reflects the influence of each filter in generating the y score.

2 $$ \alpha_{l}^{c} = \overbrace {{\frac{1}{z}\sum\limits_{i} {\sum\limits_{j} {\sum\limits_{k} {} } } }}^{{{\text{global}}\,{\text{average}}\,{\text{pooling}}}}\frac{{\partial y^{c} }}{{\underbrace {{\partial A_{ijk}^{l} }}_{\begin{subarray}{l} {\text{Gradient}}\,{\text{via}} \\ {\text{backpropagation}} \end{subarray} }}} $$

Next, the weight that influences the judgment result is calculated for each filter by multiplying $\alpha$ and $A$, and only the positive values that positively influence the classification of specific classes are extracted by applying the ReLU activation function. This is defined by Eq. (3).

3 $$ L_{Grad - CAM}^{c} { } = { }ReLU{ }\left( {\sum\nolimits_{k} {\alpha_{l}^{c} A^{l} } } \right) $$

As the size of the feature area heatmap obtained in this manner is 8 × 8 × 8, which is smaller than the size of the input voxel data, resizing is required for comparison between the two data. The voxel resizing was conducted using the SciPy library^37^. Subsequently, the maximum value of the data extracted for equal comparison between heatmaps was divided by all data to normalize it. To determine the problems in the process of features being trained at the convolution layers of the proposed network using the visual explanation technique and improve the network, Grad-CAM was conducted for Conv3D_2, Conv3D_3, Conv3D_4, and Conv3D_5 layers.

## Experiments and discussion

### System configuration

Python 3.6.9 and Tensorflow 2.4.0 libraries were employed to train the deep learning model. All the experiments were conducted on a computer with an i7-9700 K CPU (3.6 GHz), 64 GB memory, and two Nvidia Quadro RTX 5000 GPU. The gradient descent optimization used in the experiments was the Adam^38^. The batch size was 32 and 4 for 64 and 128 voxel resolutions, respectively. A total of 20% of the training datasets were used as validation datasets. To prevent overfitting, the model was trained for a maximum of 100 epochs and stopped early if the validation loss did not continue to decrease in 10 epochs. The proposed network has approximately 6.26 million parameters.

### Dataset construction

A generated parametric 3D CAD model is converted into an OBJ format that expresses a 3D object as a triangular mesh. The OBJ format consists of a number of lines; each contains a key and various values. The key on each line indicates the type of information to follow since the obj file format doesn’t require a header^39^. Next, binvox is used to convert it into a voxel within a defined grid^40^. Finally, it is converted into HDF5, a data format typically used for the management of large-scale data. HDF5, which manages data in a hierarchical data format, comprises data that include voxel information and labels that include annotation information. To reduce the data size of the HDF file, 1-bit bools and 32-bit integers were used for voxels and labels, respectively. In addition, the chunk size was divided based on the voxel resolution, and the data were compressed using the gzip method. For sufficient learning, 119,320 datasets comprising 48,000 single machining feature models and 71,320 multiple machining feature models were generated^41^. The entire dataset was divided into three sub-datasets for learning, validation, and testing. The ratio at which the datasets were divided was 75:25 for training and testing, respectively. For all datasets, shuffles and randomization were performed.

### Feature classification result

**Figure 5:**
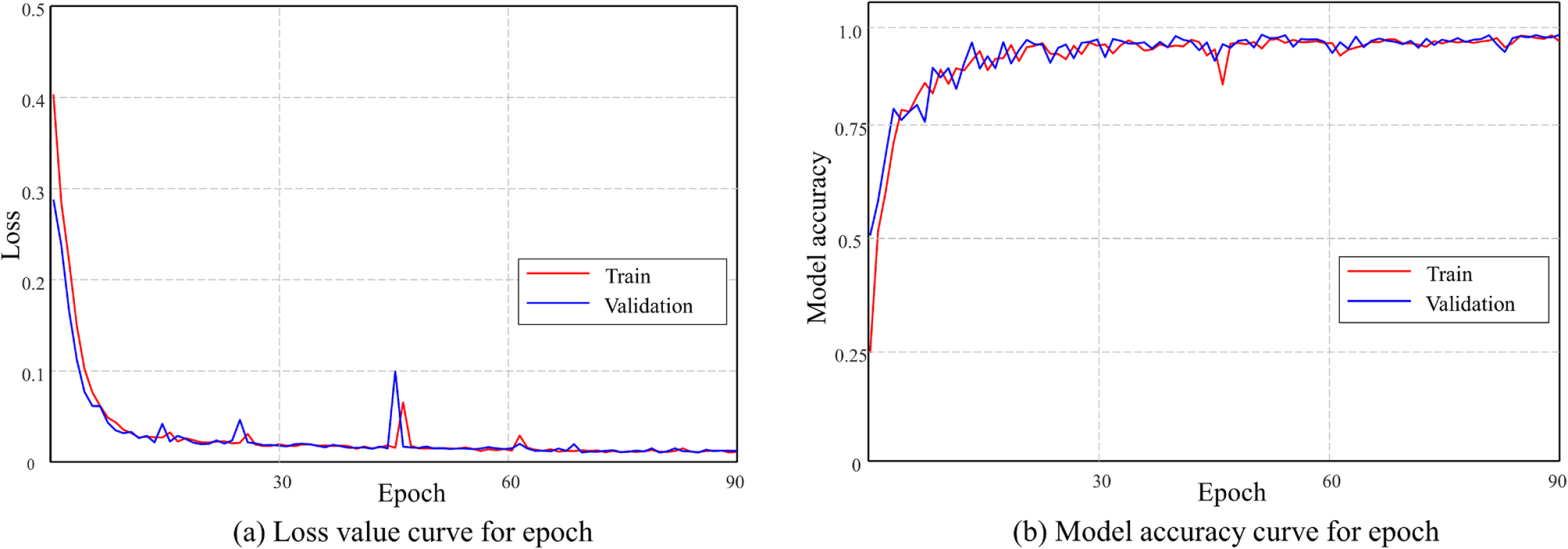
Trained model indicators of voxel size 64 for training and validation datasets.

The deep learning networks were trained using the datasets generated as described in Data generation and preprocessing“Data generation and preprocessing” section. Figure 5a shows training and validation loss for voxel size 64. As observed in the loss curve of Fig. 5a, training was performed successfully, with no occurrence of over- or under-fitting. In the case of voxel size 64, training was terminated at the 89^th^ epoch where the performance no longer improved. Figure 5b shows model accuracy by comparing the learning dataset and the validation dataset. A model accuracy is a value where the number of CAD models with all features accurately found divided by the total number of models. If every feature included in a model cannot be found, recognition is considered unsuccessful. Regarding the overall training result, the loss was 7.81 e-04, and model accuracy was 98.81% at the 79th epoch.

**Figure 6:**
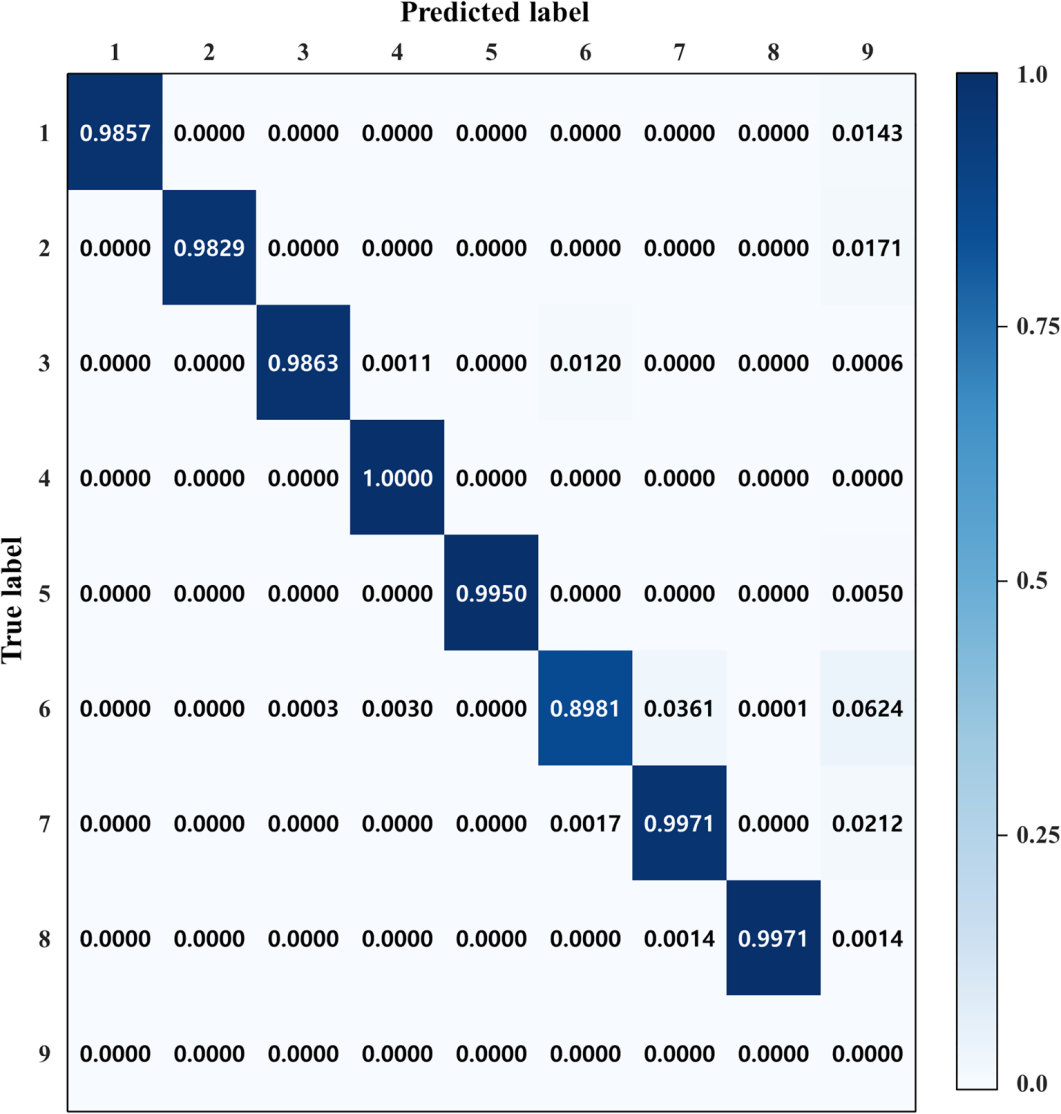
Confusion matrix of machining features in the test dataset classification.

A confusion matrix of the classification results for the single- and multi-machining features is shown in Fig. 6. Labels 1 to 9 denote fillet, chamfer, hole 1, hole 2, hole 3, pocket 1, pocket 2, pocket 3, and not-classified. To recognize the multi-features, we utilized binary cross-entropy for the loss function to classify whether or not each feature is included. We classified that the feature was included only when the prediction value exceeded 50%. Accordingly, some cases may not be classified in prediction if all prediction values were less than 50%. To distinguish this case, we added label 9, not-classified. Label 9 means that a feature was not recognized even though the feature was included in the data. The diagonal matrix excluding feature #6 (pocket 1) showed an accuracy higher than 98%. The feature #6 confused feature #7 (pocket 2) and feature #9 (not classified).

Table 6 shows the performance comparison between our network and FeatureNet. We referenced^42^ to implement FeatureNet. FeatureNet is a representative network for recognizing machined features in a 3D voxel. Furthermore, machining feature types that FeatureNet can recognize are similar to feature types defined in our dataset. Therefore, we compared the performance of our network to FeatureNet; the comparison was made using our dataset. The performance of our network was 3.01% higher than that of FeatureNet. Besides, the number of parameters of our network was 1/15 times smaller than that of FeatureNet, which benefited from the global average pooling layer.

**Table 6:** Performance comparison using our dataset between the proposed network and FeatureNet.

Network model	Classification accuracy	Number of parameters
FeatureNet^9^	95.80%	33.92 M
Our network	98.81%	2.27 M

The original data contains the most information for classification. If the stride of the first convolution layer is 2 as in FeatureNet, small machining features can be skipped. To resolve this issue, we set the stride of the first convolution layer to 1 without reducing the size of the voxel in the first convolution layer. Instead, we reduced the voxel size by placing a pooling layer after the convolution layer. In addition, FeatureNet consists of an architecture with one pooling layer after four convolution layers, but we constructed an architecture that properly mixes a convolution layer and a pooling layer. A network architecture that mixes a convolution layer and a pooling layer can not only sufficiently train but also reduce the data size.

Table 7 shows the result of ablation studies on the proposed network. Vanilla model, which is a basic model, did not apply stride 1 for the first convolutional layer nor add the 1 × 1 convolutional layer. Model 1 only applied stride 1 to the first convolutional layer, Model 2 only added the 1 × 1 convolutional layer, and Model 3 applied both. Model 1 showed poor accuracy compared to the Vanilla model. However, the performance significantly improved, outperforming the Vanilla model, when the 1 × 1 convolutional layer was added to Model 1. Tiny machining features could be effectively abstracted and transferred to the next layer by applying stride 1, instead of stride 2, to the first convolutional layer. Besides, tiny features were learned better by adding the 1 × 1 convolutional layer.

**Table 7:** Ablation study on the proposed network.

	Application of stride 1 to the first convolutional layer	Addition of the 1 × 1 Convolutional layer	Accuracy (%)
Vanilla model (FeatureNet)			95.80
Model 1	√		92.31
Model 2		√	95.38
Model 3	√	√	98.81

### Feature area detection result

**Figure 7:**
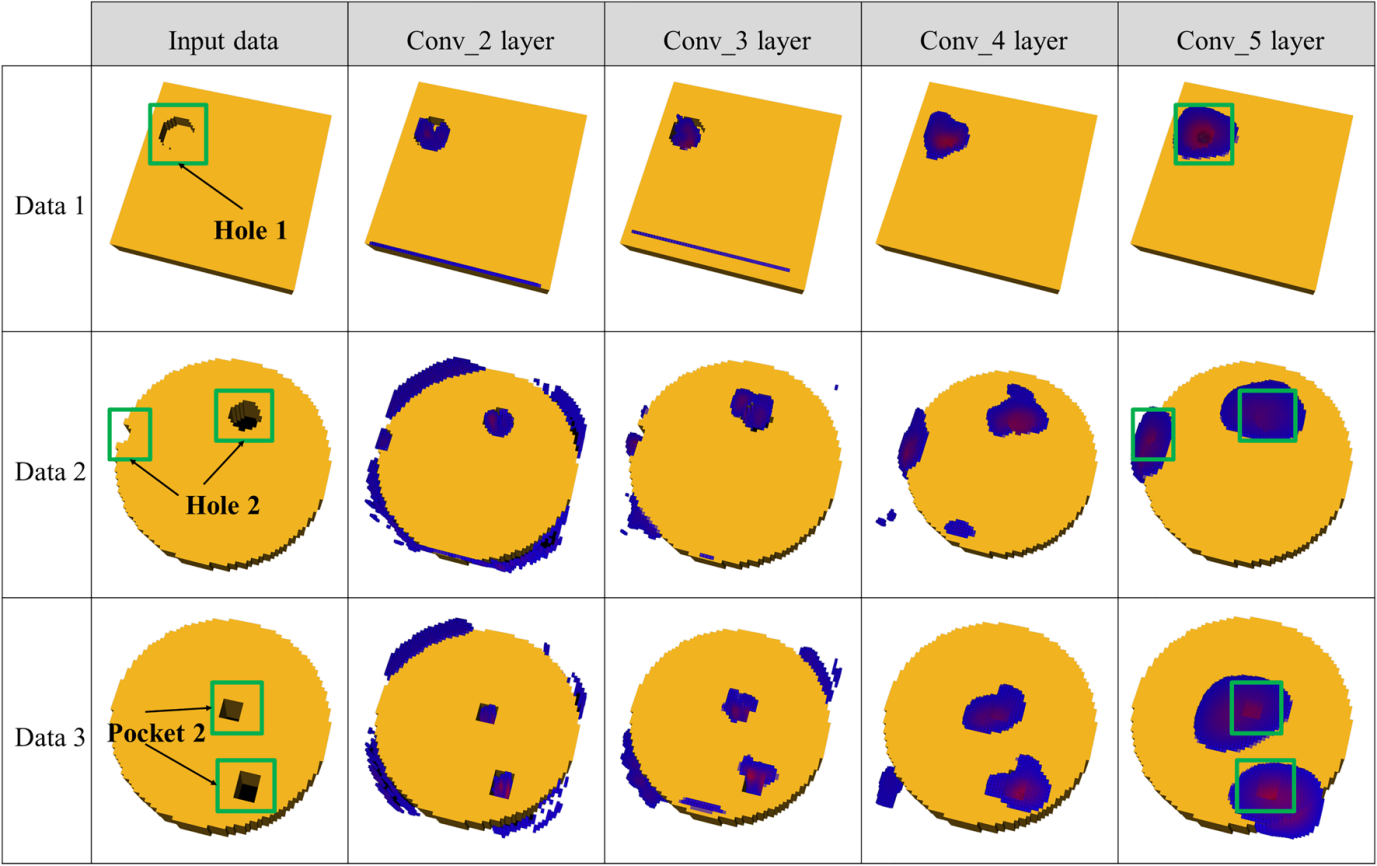
Visual explanation of the machining feature area using Grad-CAM for voxel size 64 × 64 × 64.

**Figure 8:**
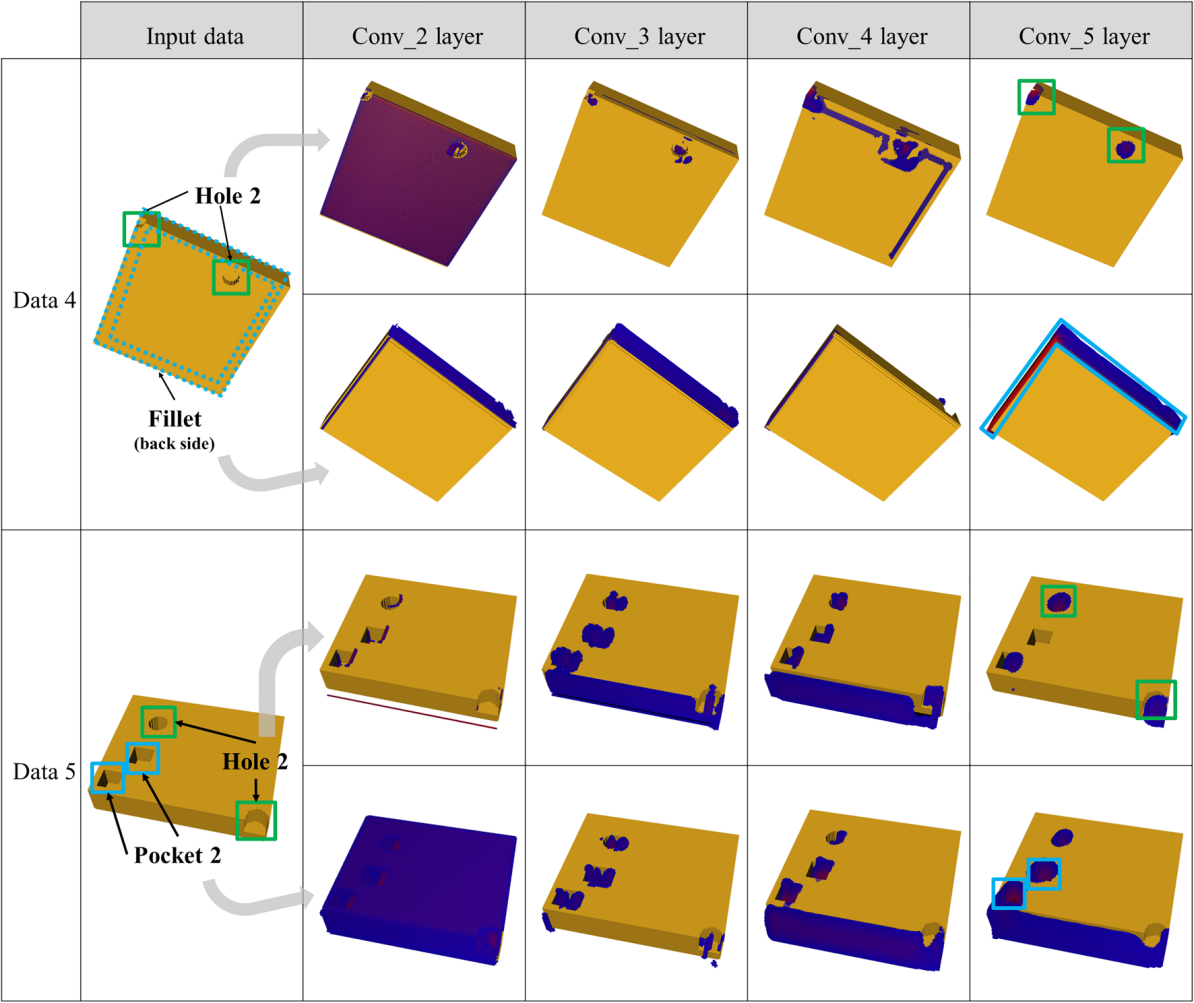
Visual explanation of the machining feature area using Grad-CAM for voxel size 128 × 128 × 128.

As mentioned in “Class activation mapping for feature area detection” section, the feature area can be estimated by applying Grad-CAM to a trained deep learning model. We analyzed the training of the deep learning model on each machining feature by applying Grad-CAM to each convolution layer. Figures 7 and 8 depict the results of applying Grad-CAM to voxel sizes 64 and 128 of the dataset, respectively. In convolution layer 2, the area detection for the boundary or candidate group of the model was conducted rather than learning the machining features.

As shown in Figs. 7 and 8, the deeper the layer becomes, the closer the area for which strong area detection is conducted is to the machining feature. In addition, the area was more accurately estimated when the voxel size was 128 × 128 × 128 than when it was 64 × 64 × 64. From blue to red, it corresponds to a higher score in the class. This meant that as the size of the voxel grew, it could include machining feature information in a smaller unit, and it could also expand the network size. However, as there is a limit in the memory that can be processed by the GPU, learning cannot be conducted if the voxel size exceeds 128 × 128 × 128.

**Figure 9:**
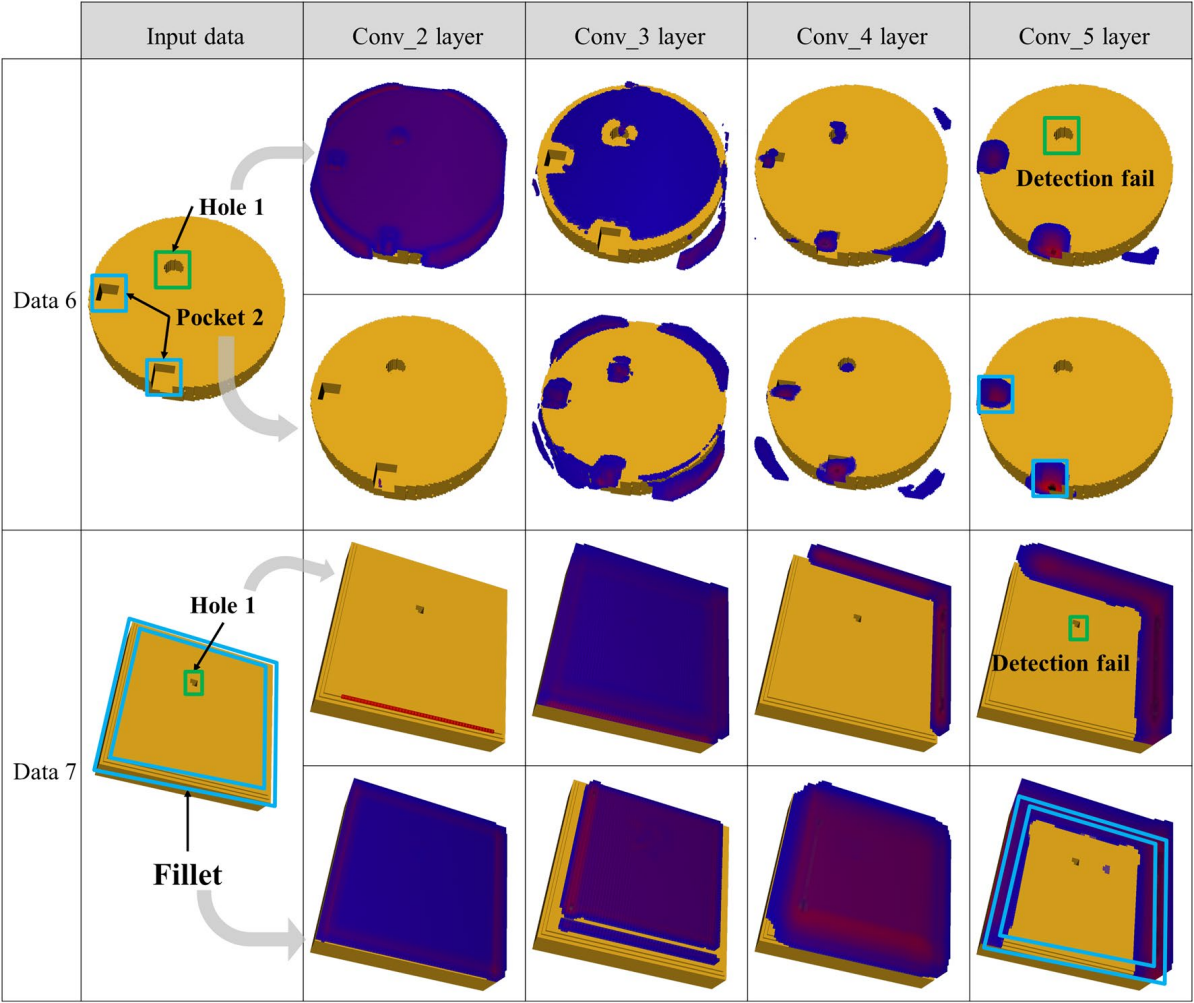
Failure case in feature area detection.

Figure 9 illustrates a failure case of area detection. When hole area detection was attempted, holes and pockets were simultaneously estimated, as shown in Fig. 9a, comprising one hole and two pockets at the initial layer, whereas only the pocket area was estimated as the layer grew deeper. However, in the classification test, the classification was successful at high probabilities of 99.99% and 99.63% for the hole and pocket, respectively. Figure 9b depicts failure in area detection of a hole of small size, 64 × 64 × 64. Although detection was performed by reducing the size of the face with a feature as the layer became deeper, the fillet area was estimated without being able to detect any hole. However, the classification was successful at high probabilities of 99.99% and 99.49% for the hole and pocket, respectively.

Grad-CAM is limited in that it cannot determine an accurate range if the same classes occur in several places^35^. In this study, although holes and pockets were defined as being separated into several independent labels as machining features in which only the numbers are different, such as one pocket (feature #6) and two pockets (feature #7) can be confused as the same class, area detection might fail. The dataset has an imbalance in the amount of data; enough data were generated uniformly for Hole 1, Hole 2, Pocket 1, and Pocket 2, but a small amount of data was generated for Hole 3 and Pocket 3. As a result, learning may not be done sufficiently, which could cause the degradation of feature area detection performance. In addition, the more the network is deepened to improve the machining feature classification performance, the surrounding context information tends not to be reflected because the receptive fields become too small in comparison to the data size. In this study, although we used GAP instead of an FC layer to reduce the complexity of the computation, the accuracy might have deteriorated because the information required for Grad-CAM area detection was lost when it passed through the GAP layer.

### Ethics approval

Not applicable.

### Consent to participate

All authors consent for participation.

## Conclusion

This study proposed a method for classifying machining features and estimating feature areas using a 3D-CNN and Grad-CAM from a 3D CAD model. To train the deep learning network, a 3D CAD model dataset that included eight machining features was built and converted into a voxel format that was used as the input data of the 3D-CNN. Our 3D CAD model dataset compositely comprised single- and multi-machining features, in contrast to existing studies that have learned only single features. Our 3D-CNN model exhibited a high classification accuracy irrespective of the machining feature type. In addition, we estimated the areas of machining features by applying Grad-CAM to the trained model and identified the problems of the network we designed.

This study confirmed the potential of deep learning in the 3D CAD field. Regarding datasets, we plan to construct a labeled dataset for each per-voxel in a 3D CAD model with machining features based on voxel segmentation in the future. In addition, we intend to resolve the Grad-CAM problem of wrongly estimating the areas for certain features by increasing the types and datasets of machining features and simultaneously improving the deep learning network.

In future work, we intend to objectively evaluate the results of Grad-CAM by putting labels for segmentation on 3D CAD models inclusive of machining features. In addition, we intend to resolve the Grad-CAM problem of wrongly estimating the areas for certain features by increasing the types and datasets of machining features and simultaneously improving the deep learning network.

## Data Availability

Dataset used in this study is available from^41^.
